# Magnetic and electronic properties unveil polaron formation in Eu$_5$In$_2$Sb$_6$

**DOI:** 10.1038/s41598-023-28711-z

**Published:** 2023-01-28

**Authors:** M. Victoria Ale Crivillero, Sahana Rößler, S. Granovsky, M. Doerr, M. S. Cook, Priscila F. S. Rosa, J. Müller, S. Wirth

**Affiliations:** 1 Max-Planck-Institute for Chemical Physics of Solids, Nöthnitzer Str. 40, 01187 Dresden, Germany; 2 Institute for Solid State and Materials Physics, Technical University Dresden, 01062 Dresden, Germany; 3 Los Alamos National Laboratory, Los Alamos, NM 87545 USA; 4 Institute of Physics, Goethe-University Frankfurt, 60438 Frankfurt (M), Germany

**Keywords:** Condensed-matter physics, Quantum physics

## Abstract

The intermetallic compound Eu$_5$In$_2$Sb$_6$, an antiferromagnetic material with nonsymmorphic crystalline structure, is investigated by magnetic, electronic transport and specific heat measurements. Being a Zintl phase, insulating behavior is expected. Our thermodynamic and magnetotransport measurements along different crystallographic directions strongly indicate polaron formation well above the magnetic ordering temperatures. Pronounced anisotropies of the magnetic and transport properties even above the magnetic ordering temperature are observed despite the Eu$^{2+}$ configuration which testify to complex and competing magnetic interactions between these ions and give rise to intricate phase diagrams discussed in detail. Our results provide a comprehensive framework for further detailed study of this multifaceted compound with possible nontrivial topology.

## Introduction

Materials in which the electronic and magnetic properties are strongly coupled hold great promise for applications in spintronics^1^. From a fundamental point of view, the underlying entanglement of magnetic, electronic and structural degrees of freedom in correlated electron materials can give rise to unexpected and often spectacular physical phenomena, e.g. high-temperature superconductivity in cuprates^2^ and colossal magnetoresistance (CMR) in magnetic semiconductors and manganites^3,4^. A characteristic of these coupled degrees of freedom is the appearance of diverse electronic phases, along with phase separation and pattern formation^5–8^. In this respect, Eu compounds are of particular interest because of their often strong exchange interaction between the charge carriers and the localized spin of Eu$^{2+}$ ions^9–11^, which can give rise to a localization of the charge carriers (of low density) around the Eu$^{2+}$ spins—so called magnetic polarons. In addition, the ground state of Eu$^{2+}$ is an isotropic $^8S_{7/2}$ configuration, limiting crystalline electric field contributions to anisotropy to higher order. In general, Eu compounds can be viewed as model systems for CMR effects and polaron formation^12–14^.

Considering Eu-based compounds, research concentrated on EuO, Eu chalcogenides and EuB$_6$, mostly because of their relatively simple crystallographic structures^11,15,16^. Recently, the topological properties of materials and their relation to crystal symmetry has been highlighted^17,18^. Specifically, materials crystallizing in nonsymmorphic space groups are prone to topological order^19,20^. Interestingly, space group *Pbam* (No. 55) fulfills these requirements^20^ including the Zintl phase Ba$_5$In$_2$Sb$_6$^21^. This drew attention to the Eu-based counterpart Eu$_5$In$_2$Sb$_6$^22^ which was known as a narrow gap semiconductor^23^. Indeed, Eu$_5$In$_2$Sb$_6$ exhibits an extraordinarily large CMR effect and signatures of polaron formation, with the possibility of axion insulating states within the antiferromagnetic regime^22^. In addition, band structure calculations for Eu$_5$In$_2$Sb$_6$ in its paramagnetic state predicted nontrivial surface states^24^ (similar to the isostructural nonmagnetic compound Ba$_5$In$_2$Sb$_6$^20^), which have not been observed^25^.

However, the complex structure of Eu$_5$In$_2$Sb$_6$ allows for three crystallographically different Eu sites^23^, and the Eu sublattices may magnetically order independently^25^. In this case, the Dzyaloshinskii–Moriya interaction needs to be taken into consideration. Independent of the crystallographic site, Eu is divalent^26^ and hence, fulfills the Zintl rule. It is noteworthy that, even though the Eu$^{2+}$ ions with orbital angular momentum $L = 0$ are isotropic, there appear to be multiple, anisotropic exchange interactions^22,25^. All this results in a complex magnetic structure which is not yet resolved experimentally.

Here we report on a comprehensive study of Eu$_5$In$_2$Sb$_6$ by magnetic, electronic transport and specific heat measurements in an effort to establish the magnetic field-temperature (*H*–*T*) phase diagrams with *H* applied along different crystallographic directions. The former two properties are highly anisotropic despite the Eu$^{2+}$ state indicating complex magnetic interactions. The temperature evolution of the studied properties establish Eu$_5$In$_2$Sb$_6$ as a rare example of a material exhibiting polaron formation in an antiferromagnet, likely also of anisotropic nature. Such electronically inhomogeneous properties in an intermetallic compound with precise electron count, i.e. in an insulating environment, provide a rich playground for possibly new quantum states, specifically when time-reversal symmetry is broken by incorporating magnetic (here rare earth) elements^27^ and materials of nonsymmorphic symmetry are investigated^19^.

## Results

### Magnetic properties

**Figure 1:**
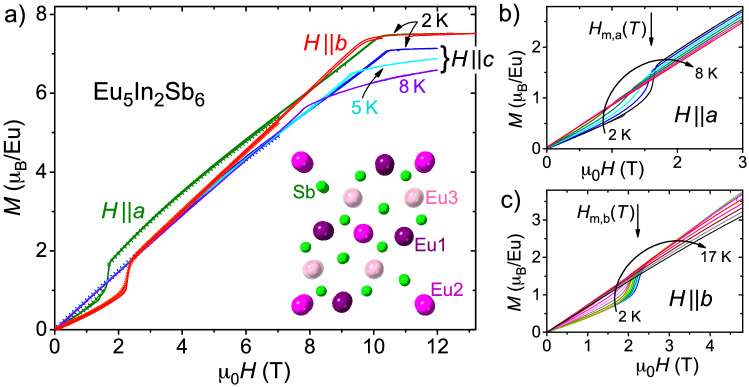
(**a**) Magnetization in dependence on magnetic field for up and down sweeps. Data at 2 K are shown for fields along all three crystallographic directions, for $H \parallel c$ also data at 5 K and 8 K are presented. Lines indicate measurements up to 14 T, markers up to 7 T. Inset: Three groups of crystallographically different Eu positions (reddish color, marked Eu1, Eu2 and Eu3) within the crystal structure of Eu$_5$In$_2$Sb$_6$ (Sb in green, In not shown) viewed along the *c*-axis. (**b**,**c**) Temperature evolution (in steps of 1 K) of magnetization curves for $H \parallel a$ and $H \parallel b$ emphasizing the metamagnetic transitions at $H_\text{m}(T)$ (arrows).

**Figure 2:**
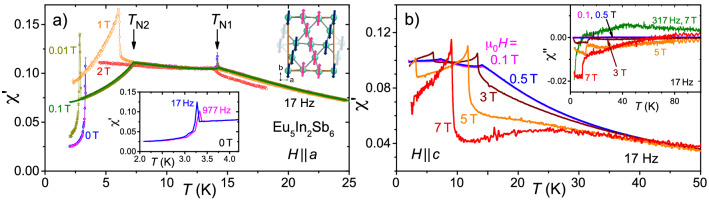
(**a**) Temperature dependent ac susceptibility $\chi '(T)$ for different magnetic fields *H* applied along the crystallographic *a*-axis illustrating the temperature shifts of the transitions with *H*. Lower inset: Slow *T*-sweep (0.1 K/min) reveals a small offset for different ac-field (2 Oe) drive frequencies for the low-*T* transition. Upper inset: suggested spin configuration. (**b**) $\chi \prime (T)$ for different *H* along the *c*-axis. Inset: Imaginary component of susceptibility, $\chi \prime \prime (T)$ for the same conditions. For comparison, $\chi \prime \prime (T)$ at 317 Hz and 7 T is shown. Different samples were measured in (**a**) and (**b**).

As already mentioned, the crystallographic structure of Eu$_5$In$_2$Sb$_6$ gives rise to three different Eu sites, cf. inset to Fig. 1a: Eu(2) and Eu(3) are surrounded by six Sb (with the octahedra around Eu(3) slightly larger than for Eu(2)), while Eu(1) has two In and seven Sb close neighbours^23^. The nearest Eu distances are between Eu(2) and Eu(3) ($d_\mathrm{Eu2-Eu3} =$ 3.7987 Å) whereas the nearest Eu to an Eu(1) is spaced $d_\mathrm{Eu1-Eu3} =$ 4.072 Å apart. Magnetic ac susceptibility $\chi '(T)$ clearly shows two magnetic transitions at $T_\text{N1} \approx$ 14.1 K and $T_\text{N2} \approx$ 7.2 K^22,28^, see Fig. 2a for $H \parallel a$, which are both suppressed in applied magnetic fields of $\mu _0 H =$ 2 T.

The magnetization curves *M*(*H*) for fields applied along the main crystallographic axes of Eu$_5$In$_2$Sb$_6$ largely conform to the behavior expected for an antiferromagnet, Fig. 1; there is an almost linear increase of *M*(*H*), specifically for $H \parallel c$, and a saturation at high fields. The saturation value of $M_\text{sat} \approx 7.0\;\mu _\text{B}$/Eu for $H \parallel c$ is consistent with the expected saturation magnetic moment of Eu$^{2+}$, $g\mu _\text{B} J = 7 \mu _\text{B}$. For $H \parallel a$ and $H \parallel b$ slightly higher values of $M_\text{sat} \approx 7.5\;\mu _\text{B}$/Eu were observed.

It is important to emphasize again the Eu$^{2+}$ state with $L = 0$. In this case, magnetic anisotropy can be attributed to anisotropic exchange, dipolar interactions or even crystalline electric fields of higher order. Hence, the clear anisotropic behavior below $T_\text{N1}$, as seen in the magnetization curves *M*(*H*), Fig. 1, for fields applied along the main crystallographic axes, is likely due to anisotropic exchange between the Eu-sites. Notably, there are magnetic transitions for $H \parallel a, b$, but not for $H \parallel c$, which indicates that the magnetic moments are (primarily) aligned within the *a*-*b* plane^25^. Figure 1b,c exhibit the disappearance of the metamagnetic transition with increasing temperature for $H \parallel a$ and $H \parallel b$, respectively. Interestingly, this transition is completely vanished at $T \gtrsim T_\text{N2}$ for $H \parallel a$, but can be followed up to $T \approx T_\text{N1}$ for $H \parallel b$. In the latter case, there is only a weak shift of the transition field $H_\text{m}$ up to $T_\text{N1}$. Furthermore, there is no notable remanent magnetization found after applying a magnetic field along any crystallographic direction, as expected for an antiferromagnetic spin configuration. However, it is essential to note that the precise magnetic structure of Eu$_5$In$_2$Sb$_6$ has not been reported yet. In the absence of detailed experimental insight, we consider a predominantly A-type antiferromagnetic order with antiparallel stacking along *c*, as suggested by density functional theory (DFT) calculations^25^, see upper inset to Fig. 2a. In all likelihood, the actual spin configuration is more complex and non-collinear, and may even support very weak ferromagnetism by spin-canting resulting from Dzyaloshinskii–Moriya interactions allowed in this low crystallographic symmetry^22,25,29^.

At even lower temperature, $T \lesssim 3$ K, there is a third transition observed only for $H \parallel a$. This transition is readily suppressed to below 2 K at fields as small as 0.1 T, and exhibits a very weak frequency dependence, see lower inset to Fig. 2a. At present, we cannot rule out In inclusions (resulting from the sample flux growth) causing this transition.

In line with the above-suggested spin configuration, $\chi \prime (T)$ exhibits a different behavior for $H \parallel c$, Fig. 2b: Here, the transition at $T_\text{N1}$ becomes ever more pronounced while shifting to lower temperature with increasing dc-field *H*. Also, the transition at $T_\text{N2}$, albeit shifted down to about 3.3 K, can still be recognized at 5 T. The shallow maximum of $\chi \prime (T \sim \mathrm {25\; K}, \mu _0 H = \mathrm {7\; T})$ and the peculiar *T*-dependence of the imaginary component of the susceptibility, $\chi \prime \prime (T)$, inset of Fig. 2b, will be discussed below.

**Figure 3:**
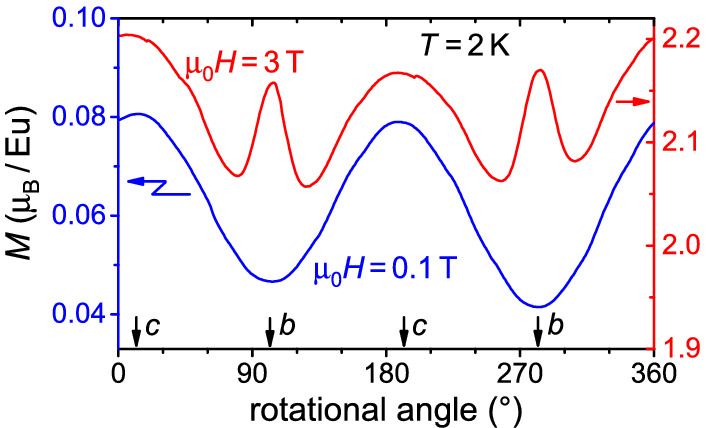
Magnetization in dependence on sample orientation with respect to *H* for two applied fields ($\mu _0 H =$ 0.1 T, 3 T) below and above $\mu _0 H_\text{m} \approx 2.2$ T at $T = 2$ K. The sample is rotated in the *b*–*c* plane, i.e. around the *a*-axis, with the alignment of the respective axis with *H* marked by arrows.

The numerous transitions and different dependencies of $\chi '$ certainly testify to an intricate response of Eu$_5$In$_2$Sb$_6$ to magnetic fields. To gain further insight, the isothermal magnetization was measured upon rotating the sample in constant applied fields. The results of one exemplary measurement is presented in Fig. 3 for fields of $\mu _0 H =$ 0.1, 3 T and at $T = 2$ K, with the sample being rotated around its crystallographic *a* axis (for more results on sample rotation see Supplementary Information, Fig. S1). At small fields (0.1 T), we find the expected two-fold symmetry: There is little response of the local moments for $H \parallel b$, where *b* is likely the easy magnetization direction^22,25^ along which the moments are already aligned in the ground state, while the moments can more easily be turned towards the *c* direction even by a small field. At $\mu _0 H =$ 3 T, the curve is just offset for a broad angular range around the *c* direction. In addition, however, there is a pronounced maximum for $H \parallel b$, which is quickly suppressed within a range of $\pm 20^{\circ }$. This observation, along with the jump in magnetization at $H_{m}$ for $H \parallel b$ shown in Fig. 1c, is a clear indication for a field-induced change in the magnetic structure (possibly a spin-flop transition). This assignment is further supported by the very weak temperature dependence of $H_{m}(T)$ and is consistent with the *b* axis being the magnetically easy direction.

There are several indications for Eu$_5$In$_2$Sb$_6$ being a candidate material for the formation of magnetic polarons near the magnetic ordering temperature, ranging from colossal magnetoresistance^22^ and piezoresistance^30^ to characteristic changes of the Eu$^{2+}$ electron spin resonance (ESR)^31^. Here, magnetic polarons refer to magnetically ordered clusters within which conduction electrons are localized via strong exchange interaction with the 4*f* moments and giving rise to spin-polarization^32^. The $\chi \prime (T)$ data for small applied field exhibit a tiny deviation from Curie–Weiss behavior below about 180 K, and a more obvious one around 50–60 K (see Supplementary Information, Fig. S2) which was argued to be caused by the formation of magnetic polarons and correlations between them, respectively. Very likely, the polarons grow in size with increasing applied field such that magnetic correlations become more pronounced. As seen in Fig. 2b, this gives rise to an ever stronger reduction of $\chi \prime (T)$ (and hence, stronger deviations from Curie–Weiss behavior) with increasing *H* for temperatures above $T_\text{N1}(H)$. For magnetic fields as high as $\mu _0 H =$ 7 T, even a shallow maximum of $\chi \prime (T)$ is observed. We emphasize that differences in $\chi \prime (T)$ for different applied fields are clearly seen below about 45 K, i.e. where magnetic polarons start to significantly influence the sample properties (see also results for $\chi _\text{dc}$ in Supplementary Information, Fig. S3). This picture is corroborated by the imaginary component of the susceptibility, $\chi \prime \prime (T)$, presented in the inset of Fig. 2b. $\chi \prime \prime$ is related to dissipative magnetic processes and is typically zero in antiferromagnetic phases. Interestingly, for large enough applied fields, $\chi \prime \prime (T \lesssim 50 \text{K})$ is finite, indicating ferro- or ferrimagnetic contributions, and negative, consistent with an inhomogeneous state of magnetization^33^. Moreover, $\chi \prime \prime$ is positive, if measured at 317 Hz and 7 T, emphasizing a changed response to higher drive frequencies.

The behavior just described is also observed when the magnetic field is applied along the crystallographic *b* axis. In comparison to other compounds for which a magnetic polaron scenario is considered, the phenomenology found in Eu$_5$In$_2$Sb$_6$ is consistent with findings for EuB$_6$ (see, e.g., susceptibility data in Ref.^34^), but differs from the one reported in Sr doped LaCoO$_3$ samples^35^. In the latter case, the spin-glass behavior of ferromagnetic clusters embedded in a non-ferromagnetic matrix is thought to be revealed by a frequency-dependent cusp in the ac susceptibility data.

### Heat capacity

**Figure 4:**
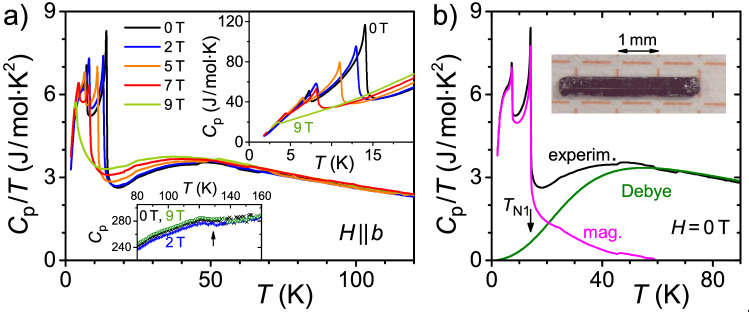
(**a**) Specific heat divided by temperature, $C_p/T$, in dependence on *T* for applied fields $H \parallel b$ up to 9 T. The insets show enlarged views of $C_p(T)$ within certain temperature ranges (in units of J/mol K). (**b)** $C_\text{ph}$ contribution from a Debye model (green curve) fitted to the experimental $C_p(T)$-data (black) within 60 K $\le T \le$ 200 K and used to estimate $C_\text{mag}/T$ (magenta). Inset: photograph of one representative Eu$_5$In$_2$Sb$_6$ sample with the long dimension corresponding to the *c* axis.

Results of the measurements of the specific heat $C_p (T,H)$ or different magnetic fields $H \parallel b$ are presented in Fig. 4. Here, the main panel shows $C_p / T$ as a function of *T*, while the insets zoom into $C_p$-data within two temperature ranges of interest. The two transitions at $T_\text{N1}$ and $T_\text{N2}$ as well as their suppression in magnetic field are clearly revealed. Importantly, the impact of applied magnetic fields can be recognized up to temperatures of about 60 K whereas at higher temperatures there are no obvious differences between the data obtained at different magnetic fields. The experimentally observed zero-field $C_p/T$-data exhibit a pronounced maximum at $T_\text{max} \sim$ 45 K which shifts to lower temperatures with increasing magnetic field ($T^{9T}_\text{max} \sim$ 37 K). Consequently, we will relate this maximum to the magnetic properties of Eu$_5$In$_2$Sb$_6$.

To analyse $C_p$, magnetic, electronic and phonon contributions to the specific heat are considered, $C_p(T,H) = C_\text{mag} + C_\text{el} + C_\text{ph}$. Here, $C_\text{el}=\gamma \,T$ and $C_\text{ph}$ are described by a linear-in-*T* and a Debye model, respectively. Given the number of parameters, the above description of $C_p$ cannot unambiguously be fitted to our experimental data. Therefore, $(C_\text{el} + C_\text{ph})$ was fitted to the experimental data only for 60 K$\,\le T \le \,$200 K, i.e. in a *T*-range above the temperature below which differences for different magnetic fields were seen. The fit yields a Debye temperature of $\theta _\text{D}=$ 197 K (green curve in Fig. 4b) and a vanishing value for $\gamma$. Using these fit results to subtract the corresponding contributions from the experimental data in the low-temperature range, $C_\text{mag}/T = C_p/T - C_\text{ph}/T$ shown as magenta curve in Fig. 4b, reveals a significant magnetic contribution to the specific heat even above $T_\text{N1}$. A very similar behavior was observed for a second set of samples measured up to 280 K to improve on the Debye fit, see Supplementary Information Fig. S4. Integrating $C_\text{mag}/T$ up to $T = 15$ K, i.e. just above $T_\text{N1}$ but well below $T_\text{max}$ yields about 80% of the expected magnetic entropy $S_\text{mag} = 5R\ln (2S+1) \approx$ 86.4 J/mol K (where *R* and *S* are the gas constant and the spin momentum of Eu$^{2+}$, respectively), i.e. the integration has to be carried out up to higher temperatures to recover the full magnetic entropy. This inference holds despite the uncertainty of our analysis as well as for our data in magnetic field (see Supplementary Information Fig. S4) and reinforces our conclusion above that the feature related to $T_\text{max}$ is magnetic in nature.

Near $T_\text{max} \sim$ 45 K a strong deviation of the susceptibility from a Curie–Weiss law was observed (Supplementary Fig. S2) which was previously ascribed to the onset of interactions between polarons^22^. Following this interpretation, we assume this maximum to result from a *field-dependent* Schottky-like anomaly. Indeed, there are examples of such Schottky-like anomalies, where the temperature $T_\text{max}$ decreases with increasing magnetic field^36^. Also, a low-temperature Schottky anomaly in LaCoO$_3$ has been previously linked to magnetic polarons^37^. If an interpretation of $T_\text{max}$ based on a polaron-interaction scenario is correct, the *formation* of polarons is expected to take place at significantly higher temperatures. Here we note that there is a small hump observed in $C_p(T)$ at $T_\text{hump} \sim$ 130 K, lower inset to Fig. 4a, a temperature which is somewhat lower compared to the one expected for polaron formation from susceptibility, Supplementary Fig. S2, and Refs.^22,31^.

A Schottky contribution $C_\text{Sch}$ to $C_p$ is associated with a two level system^38^. We speculate that such a two-level scenario may result from ferromagnetic (fm) correlations related to the magnetic polarons on the one hand, and antiferromagnetic interactions between Eu ions on the other hand. The latter are expected to prevail at lower temperature near $T_\text{N1}$ while fm correlations are likely the dominant ones at higher temperatures, where they are expected to be stabilized in magnetic fields. Indeed, assuming a lattice background independent of magnetic field and fitting the specific heat data well above $T_\text{N1}$ with a Debye term and a magnetic field-dependent Schottky contribution reveals a systematic increase of the level splitting from $\delta \sim$ 2.2 meV at $\mu _0 H =$ 0 to about 4.5 meV at 9 T, while at the same time the magnitude of the magnetic Schottky contribution shows a $\sim 30$ % decrease with increasing field. This explains the apparent shift of $T_\text{max}$ in $C_p/T (T)$ to lower temperatures with increasing field.

Albeit a Schottky anomaly linked to polaron interaction is consistent with the observed temperature evolution of $C_p$, we cannot exclude alternative explanations. For instance, a temperature well above $T_\text{N1}$ required to gain the full magnetic entropy might also result from some partial ordering due to magnetic (possibly geometric) frustration between different Eu sites^25^. We wish to stress, however, that—independent of any fitting or interpretation—there is a considerable amount of magnetic contribution to $C_p$ found at temperatures well above $T_\text{N1}$.

### Magnetic phase diagrams

In order to gain more insight into the magnetic behavior we construct *H*–*T* phase diagrams for the different crystallographic directions making use of our magnetization, susceptibility and specific heat data. The resulting phase diagrams for $H \parallel a$ and $H \parallel c$ are qualitatively similar, with *c* clearly being the magnetically hard direction. We note that for $H \parallel a$ the jump of *M*(*H*) at 2 K and $\sim$ 1.6 T (Fig. 1b) is indicative of a metamagnetic transition, similar to the observation for $H \parallel b$ (Fig. 1c). However, in contrast to the latter case, the transition for $H \parallel a$ is readily suppressed near $T_\text{N2}$. This makes an assignment of this transition for $H \parallel a$ to $T_\text{m}$ or $T_\text{N2}$ difficult. Moreover, only in case of $H \parallel a$ is there a slight change of slope of *M*(*H*) near 5 T, Fig. 1a, and a third transition seen in $\chi \prime (T)$ at $\mu _0 H \le$ 0.1 T, Fig. 2a.

**Figure 5:**
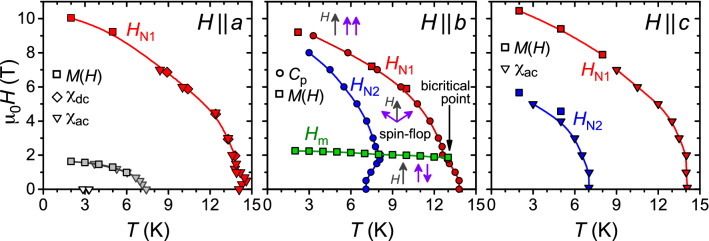
*H*–*T* phase diagrams for magnetic fields aligned along different crystallographic directions, left: $H \parallel a$, center: $H \parallel b$, right: $H \parallel c$, as extracted from ac and dc susceptibility $\chi$, magnetization *M* and specific heat $C_p$ measurements. Violet arrows depict a possible magnetization orientation with respect to magnetic field (gray arrows). To enable comparison, sample demagnetization effects are taken into consideration.

A more complex behavior is found for the *b* axis, i.e. the magnetically easy direction, where a field-induced change in the magnetic structure (likely a spin-flop transition) at $H_\text{m}$ is observed. This gives rise to a bicritical point, where $H_\text{m}(T)$ terminates at $H_\text{N1}(T)$. Here, $T_\text{N1}(H)$ exhibits the characteristic kink at the bicritical point^39^. The non-monotonic *H*-dependence of $T_\text{N2}(H)$ indicates a stiffening of the ordering taking place at $T_\text{N2}$ for fields below the spin-flop transition, while above $H_\text{m}$ the ordering is successively suppressed with increasing field. One may speculate that a similar interplay of exchange interactions is at play for $T_\text{N1}(H)$ at very small fields $H \parallel a$. All these observations point to a complex magnetic exchange and ordering mechanism in Eu$_5$In$_2$Sb$_6$ as can be expected from the crystallographic structure.

### Resistivity measurements

**Figure 6:**
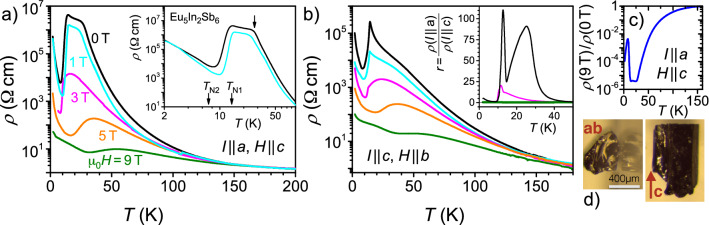
Resistivity $\rho (T)$ in dependence on temperature for current *I* along different directions: (**a**) $I \parallel a$ and $H \parallel c$ and (**b**) $I \parallel c$ and $H \parallel b$. Inset in (**a**): Zoom into the low-*T* range on a logarithmic *T*-scale. At zero field, a kink is observed around 27 K (arrow). Inset in (**b**): Ratio *r* of resistivities for the different configurations shown in (**a**) and (**b**). The zero-field maximum near 27 K is suppressed with field. At $H = 9$ T, *r* is of order 0.3 (green curve). All panels share the same color code for magnetic fields. (**c**) Ratio of resistivities measured at fields of 9 T and 0 T, as shown in (**a**). (**d**) Photographs of the sample, scale bar 400 $\upmu$m.

Electronic transport in general, but specifically in a material as complex as Eu$_5$In$_2$Sb$_6$ can be influenced by a number of factors (this may also include In inclusions originating from sample growth). Indeed, resistivity measurements carried out on different samples exhibited somewhat different $\rho (T )$-results, in particular for $T < T_\text{N1}$ (compare results shown in Fig. 6 and Supplementary Information Fig. S5). Therefore, resistivity data were not included in constructing the phase diagrams of Fig. 5. Rather, we make use of the insight gained from other measurements so far when discussing the electronic transport results in the following.

In Fig. 6 results of the transversal resistivity $\rho (T,H)$ are compared for currents *I* along the *a* and the *c* direction, respectively. Clearly, the strong anisotropy of Eu$_5$In$_2$Sb$_6$ is also seen in $\rho (T)$, specifically in zero field. For $I \parallel a$ the resistivity $\rho _{I\parallel a}(T)$ rises much more rapidly upon cooling than $\rho _{I\parallel c}(T)$. This behavior (also seen in Supplementary Fig. S5) may be related to the crystal structure of Eu$_5$In$_2$Sb$_6$ which is characterized by infinite [In$_2$Sb$_6$]$^{10-}$ ribbons oriented parallel to the crystallographic *c* axis^23^ and hence, favoring transport for $\rho _{I \parallel c}(T)$. Near 27 K, $\rho _{I\parallel a}(T)$ exhibits a kink into a much less-steep behavior [see arrows in insets to Fig. 6a and Supplementary Fig. S5] before dropping by several orders of magnitude below $T_\text{N1}$. For $I\parallel c$, a smaller hump is detected, in agreement with Ref.^22^. In consequence, the ratio of resistivities, $r = (\rho _{I \parallel a})/(\rho _{I\parallel c})$ measured for the two different current directions exhibits a pronounced maximum near 27 K, inset of Fig. 6b. The second, sharper maximum at 12.7 K is likely related to the antiferromagnetic order at $T_\text{N1}$. Larger spin fluctuations can be expected in the plane perpendicular to the magnetically hard *c* direction, increasing $\rho _{I \parallel a}$, an effect that quickly subsides for decreasing *T*, i.e. away from the magnetic transition.

As mentioned above, $\chi \prime (T)$ exhibits a deviation from Curie–Weiss behavior below about 50 K (Supplementary Fig. S2). Therefore, the rise of *r*(*T*) for $T \lesssim 50$ K is very likely linked to the magnetic behavior of Eu$_5$In$_2$Sb$_6$, in line with the specific heat results discussed above. More specifically, ellipsoidal polarons have been suggested for Eu$_5$In$_2$Sb$_6$ which start to interact below about 50 K^30^. We speculate that these ellipsoidal polarons are more extended perpendicular to the magnetically hard *c* direction and strongly interact below $\sim$ 27 K, possibly causing the plateau-like behavior of $\rho _{I\parallel a}(T)$. A further growth of the polarons upon decreasing *T* also increases interaction along the *c* direction resulting in the drop of *r*(*T*) below about 25 K. Applying magnetic fields is expected to level out inhomogeneities in the magnetic state of Eu$_5$In$_2$Sb$_6$. Our finding of a significantly suppressed *r*(*T*) already at 3 T, inset of Fig. 6b, strongly supports such a polaron scenario. In fields as high as 9 T magnetic inhomogeneities are further smoothed out, and *r*(*T*) exhibits an almost flat behavior.

As expected in a polaron scenario, large MR effects are observed^22^, especially in a temperature range $T_\text{N1} \lesssim T \lesssim 27$ K. This can be inferred from the ratio of resistivities $\rho (9\,\text{T}) / \rho (0\,\text{T})$, plotted in dependence on *T* for $I \parallel a$ in Fig. 6c. This ratio reaches values well below 10$^{-5}$, corresponding to a CMR [defined as $(\rho (H) - \rho (0)) / \rho (0)$] of more than − 99.999%. The anisotropy of $\rho$ in Eu$_5$In$_2$Sb$_6$ is also reflected in the MR: For $I\parallel c$, the ratio $\rho (9\,\text{T})/\rho (0\,\text{T})$ goes only down to $\sim \! 1.6 \times 10^{-4}$ close to $T_\text{N1}$.

One characteristic of materials exhibiting polaron formation is a low carrier concentration *n* such that energy gain by carrier localization is achieved^32^. Here, Hall measurements are called for. However, the determination of *n* is straightforward only if inhomogeneous magnetic states and spurious contributions to the Hall signal are avoided. Within this limitation (i.e. for $\mu _0 H \ge$ 7 T where a linear Hall response was observed) we obtain $n \approx 1.8 \times 10^{17}$ cm$^{-3}$ at $T =$ 100 K assuming a single (hole-like) band (see Supplementary Information, Fig. S6). This is the same order of magnitude as the carrier density $n \sim 1 \times 10^{17}$ cm$^{-3}$ reported^22^ for $T =$ 300 K. Note that our value of *n* corresponds to about 0.02 carriers per Eu site, a number very similar^40^ to the one found for EuB$_6$ which is a prototypical material for polaron formation^13,41^.

## Conclusions

The CMR effect observed in Eu$_5$In$_2$Sb$_6$ is very likely related to the formation of magnetic polarons, similar to EuB$_6$ or the doped manganites. However, there are also marked differences: (i) As seen from Fig. 6 (c) the MR in Eu$_5$In$_2$Sb$_6$ is always negative whereas the MR in EuB$_6$ and doped manganites turns positive below the magnetic ordering temperature (for discussions, see Refs.^3,42)^. Likely, this is related to the ferromagnetic ordering in the latter two materials while Eu$_5$In$_2$Sb$_6$ orders antiferromagnetically. Therefore, Eu$_5$In$_2$Sb$_6$ provides a rare opportunity to study magnetic polaron formation in an antiferromagnetic environment. (ii) The large MR effects in Eu$_5$In$_2$Sb$_6$ extend to comparatively high temperatures. The resistivity is still suppressed by an *order of magnitude* in $\mu _0 = H$ 9 T at around 75 K, i.e. at more than six times $T_\text{N1}$. By comparison, the polaron formation in EuB$_6$ only *sets in* at around 35 K^41,43^, which corresponds to about three times the ferromagnetic ordering temperature. (iii) The highly anisotropic magnetic and transport properties of Eu$_5$In$_2$Sb$_6$ are certainly related to its complex crystallographic structure. This is in line with the observation of highly anisotropic electronic transport properties in the manganites with layered perovskite structure^44^.

The latter, anisotropic properties stem from anisotropic exchange interactions, given the magnetically isotropic Eu$^{2+}$ ground state configuration $^8 S_{7/2}$. Earlier spin-resolved band structure calculations suggested a spin configuration with ferromagnetically coupled Eu moments in the *ab* plane, which are alternatingly stacked in an antiparallel staggered order^25^. Our angular dependent magnetization measurements support such a picture. In particular, a spin-flop transition for $H \parallel b$ indicates a preferred alignment of magnetic momentums along the *b* direction.

The here established phase diagrams can guide future exploration of this complex material. Clearly, the magnetic structure needs to be resolved further, including a possible weakly ferromagnetic component resulting from spin canting due to Dzyaloshinskii–Moriya exchange. The insights provided here are essential for an evaluation of the surface topology in this nonsymmorphic material. In particular, Eu$_5$In$_2$Sb$_6$ may provide a test case for studying the impact of electronic inhomogeneities on topology^45,46^.

## Methods

Single crystalline samples Eu$_5$In$_2$Sb$_6$ were grown by a combined In-Sb self-flux technique^22^. The crystallographic structure was confirmed by X-ray diffractometry. For sample orientation a real-time Laue X-ray system was employed (Laue-Camera GmbH). For magnetization measurements up to magnetic fields of 14 T a vibrating sample magnetometer (VSM, Oxford Instruments Inc.) was utilized. Magnetic measurements up to 7 T were also conducted using a magnetic property measurement systems (MPMS3, Quantum Design Inc.). If not noted otherwise, susceptibility was measured with an applied ac field of 10 Oe after cooling in zero applied field (ZFC). The angle dependence of magnetization was measured by means of a horizontal rotator inside an MPMS-XL, Quantum Design Inc. Specific heat was measured using a physical property measurement system (PPMS, Quantum Design Inc.) equipped with a calorimeter that utilizes a quasi-adiabatic thermal relaxation technique. Electronic transport was investigated using the same PPMS system. In an effort to accurately measure the high sample resistances at low temperatures, an external, lock-in-based circuitry hooked up to the PPMS was implemented. In addition, to enable electronic transport measurements, a somewhat thicker sample was used (Fig. 6d).

## Supplementary Information

Supplementary Information.

## Data Availability

Full sets of crystallographic data generated and/or analysed during the current study have been deposited with the joint CCDC and FIZ Karlsruhe deposition service. The data can be obtained free of charge from The Cambridge Crystallographic Data Centre via https://www.ccdc.cam.ac.uk/structures citing deposition number CSD 2214610. All other data generated and analysed during this study are included in this published article (and its supplementary information files) and/or are available from the corresponding author on reasonable request.
