# Supplementary material for: Magnetic and electronic properties unveil polaron formation in Eu5In2Sb6
Source: Sci Rep. 2023 Jan 28;13:1597. doi: 10.1038/s41598-023-28711-z (PMC9884272; doi:10.1038/s41598-023-28711-z)
Supplement: Supplementary file 1 — Supplementary Information. [file 41598_2023_28711_MOESM1_ESM.pdf]

Supplementary Information
Magnetic and electronic properties unveil polaron formation in $Eu_5In_2Sb_6$

M. Victoria Ale Crivillero,[1] Sahana Rößler,[1] S. Granovsky,[2] M. Doerr,[2] M. S. Cook,[3] Priscila F. S. Rosa,[3] J. Müller,[4] and S. Wirth[1]

[1] *Max-Planck-Institute for Chemical Physics of Solids, Nöthnitzer Str. 40, 01187 Dresden, Germany*
[2] *Institut für Festkörper- und Materialphysik, Technische Universität Dresden, D-01062 Dresden, Germany*
[3] *Los Alamos National Laboratory, Los Alamos, NM 87545, USA*
[4] *Institute of Physics, Goethe-University Frankfurt, 60438 Frankfurt (M), Germany*

(Dated: January 18, 2023)

Content:

I. Angular dependence of Magnetization

II. Additional measurements of magnetic properties

III. Heat capacity analysis

IV. Additional resistivity data

V. Hall measurements

I. ANGULAR DEPENDENCE OF MAGNETIZATION

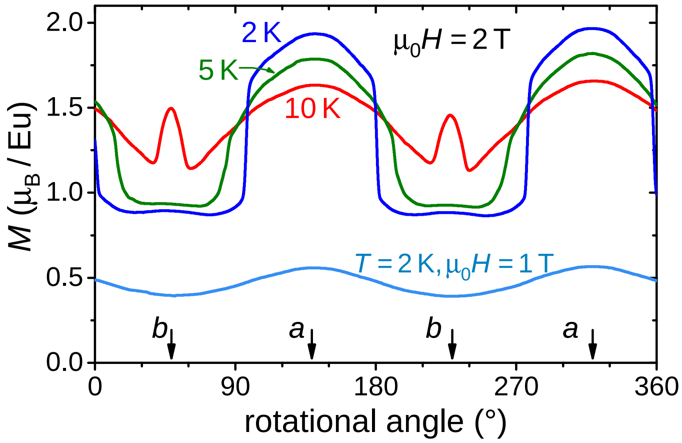

FIG. S1. Magnetization in dependence on sample orientation upon rotation around its c axis, i.e. in the a-–b plane. Data measured at constant magnetic field, here $\mu_0 H = 2$ T, allow for a direct comparison to $M(H)$, Fig. 1 of the main text. At $T = 2$ K, this field is beyond the jump in $M(H)$ for $H \parallel a$ but below the jump for $H \parallel b$ (cf. Figs. 1(b), (c) of the main text). This is clearly reflected in the two-fold symmetry of the orientation dependence of M (blue curve). A similar behavior is observed at 5 K (green curve). At 10 K, the field of 2 T induces a spin-flop transition for $H \parallel b$ (cf. phase diagrams in Fig. 5 of the main text) and an additional pronounced maximum is observed for sample orientations with b close to the direction of H, very similar to those in Fig. 3 of the main text. For comparison, a curve obtained at $\mu_0 H = 1$ T and $T = 2$ K is also shown (light blue) clearly indicating a two-fold symmetry.

II. ADDITIONAL MEASUREMENTS OF MAGNETIC PROPERTIES

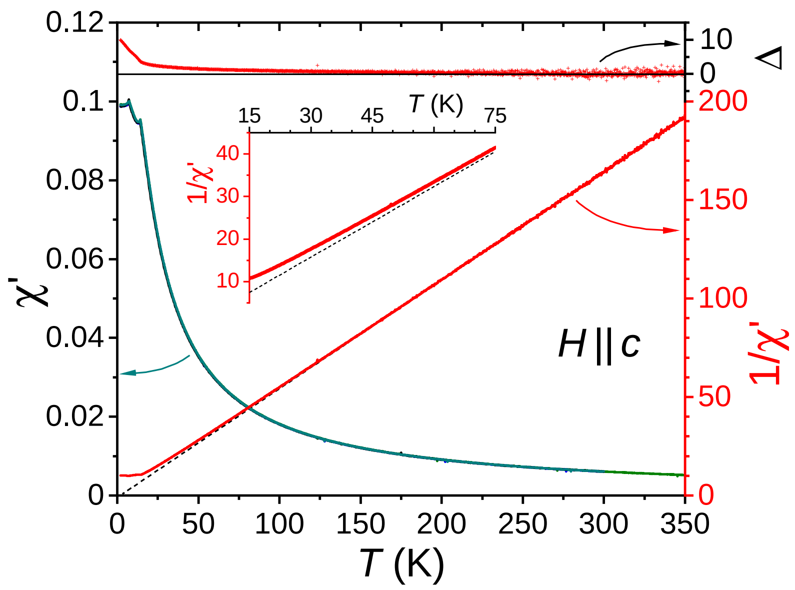

FIG. S2. AC susceptibility $\chi'(T)$ as measured up to 350 K with $\mu_0 H = 0.1$ T applied parallel to c; in addition a 2 Oe ac field of 3, 170, 317 and 517 Hz was applied without visible differences in $\chi'(T)$. Also shown is the inverse susceptibility, $1/\chi'(T)$, as well as a linear fit of the high-temperature data (200 K $\leq T \leq$ 350 K) yielding a positive intercept at $\theta \sim 2.2$ K and an effective moment of 7.8 μ_BEu^{-1} (Hund's rule moment of Eu^{2+} is 7.94 μ_BEu^{-1}). The inset exhibits a zoom into the $1/\chi'(T)$-data for 15 K $\leq T \leq$ 75 K, i.e. within the T-range of interest for polaron formation. The upper red data points reveal the quality of the fit, with Δ denoting the difference between the $1/\chi'(T)$-data and the linear fit.

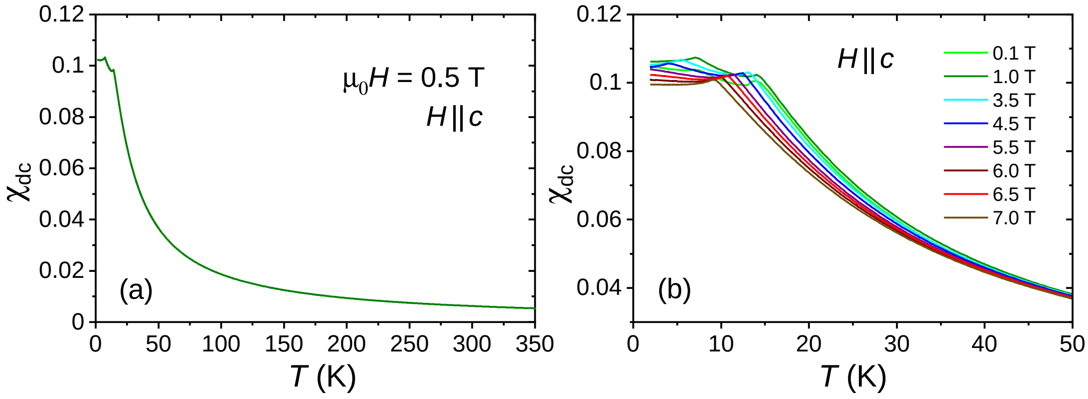

FIG. S3. DC susceptibility $\chi_{dc}(T)$ measured for different samples and with magnetic fields applied parallel to the crystallographic c direction. (**a**) Full temperature range up to 350 K with $\mu_0 H = 0.5$ T. (**b**) $\chi_{dc}(T)$ for different magnetic fields and for temperatures up to 50 K.

III. HEAT CAPACITY ANALYSIS

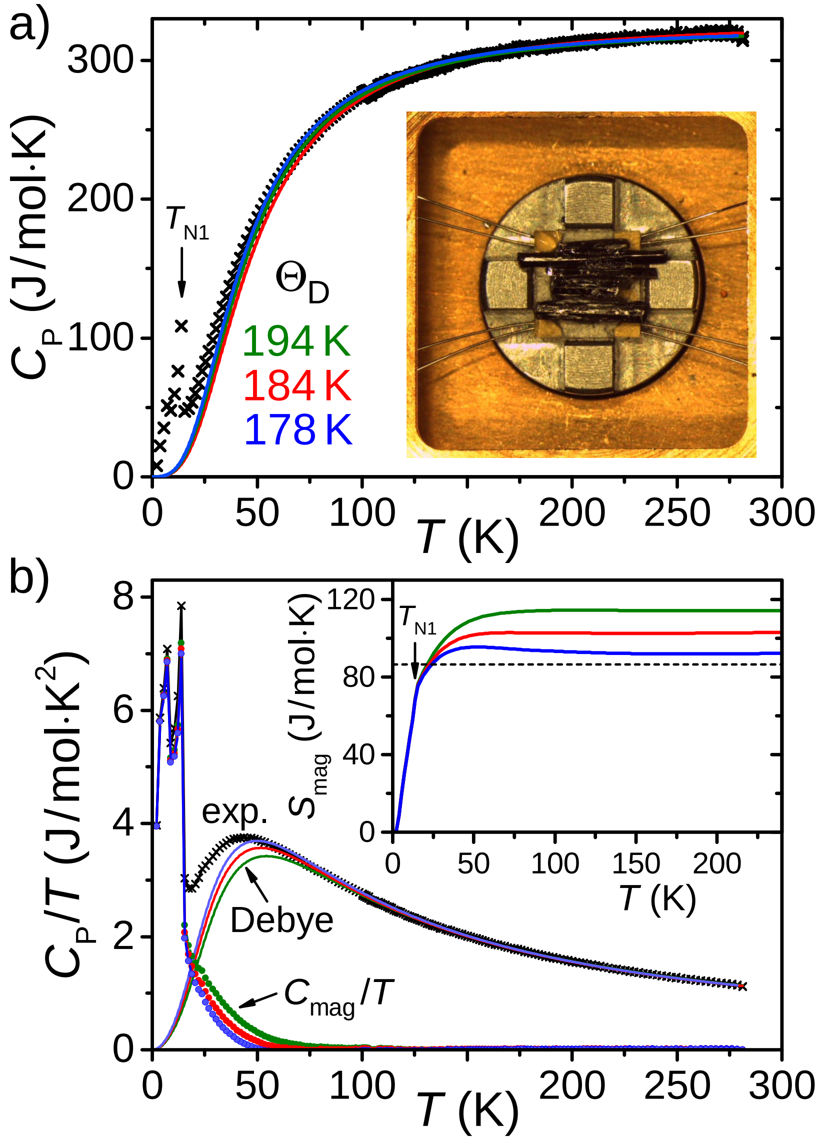

FIG. S4. Detailed analysis of the specific heat data. (**a**) C_p data (markers) as measured on a set of five samples (shown in the inset, mounted on PPMS heat capacity puck, square cutout $\sim 9.5 \times 9.5$ mm^2) to optimize accuracy. Measurements were conducted up to 280 K to enlarge the Debye fit range compared to the measurements reported in the main text (a Debye model was used to describe the phonon contribution $C_{\rm ph}$ to C_p). Results of fits for three different Debye temperatures $\theta_{\rm D}$ (red–194 K, green–184 K, blue–178 K) are included as lines. The obtained $\theta_{\rm D}$ depends mostly on the lower limit of the temperature range considered in the respective fit. (**b**) Same data as in (a) presented as C_p/T. In addition, the differences $C_{\rm mag}/T = (C_p - C_{\rm ph})/T$ are shown by markers of colors corresponding to Debye fits with different $\theta_{\rm D}$. Irrespective of the exact value of $\theta_{\rm D}$ there is a considerable contribution $C_{\rm mag}/T$ well above $T_{\rm N1} \approx 14.1$ K. Inset: magnetic entropy $S_{\rm mag}$ estimated from $C_{\rm mag}/T$ by assuming different $\theta_{\rm D}$ in $C_{\rm ph}$ [same color code as in (a)]. All curves overestimate the value of $5R\ln(2S+1) \approx 86.4$ J/mol K (dashed line) expected at high temperature. The clear *decrease* of $S_{\rm mag}$ above $\sim$50 K for $\theta_{\rm D} = 178$ K (blue line) indicates that the fitted phonon contribution $C_{\rm ph}/T$ exceeds the experimental values C_p/T. Note that $S_{\rm mag} \approx 30.3$ J/mol K at $T_{\rm N2} \approx 7.2$ K, a value that is influenced only very little by the choice of $\theta_{\rm D}$. Hence, it is unlikely that the Eu(2) ions order at $T_{\rm N2}$ as they account for only 1/5 of the Eu.

IV. ADDITIONAL RESISTIVITY DATA

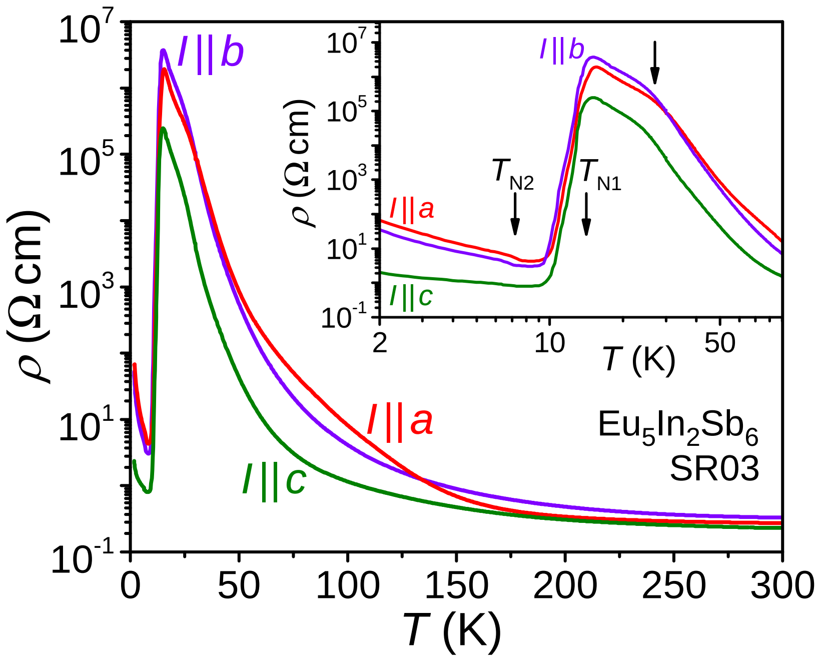

FIG. S5. Temperature dependence of the resistivity $\rho(T)$ measured on a different sample $Eu_5In_2Sb_6$ (denoted SR03). Results for currents I applied along the main crystallographic directions a, b and c are presented. The values at high temperature are comparable to those of the sample presented in the main text (Fig. 6), and in both cases resistivities for $I \parallel a$ exceed those for $I \parallel c$ (by about an order of magnitude just above T_{N1}). However, in the antiferromagnetic regime $T < T_{N1}$, the $\rho(T)$-data of the sample presented here are significantly lower. Moreover, the kink observed at around 27 K (marked by an arrow in the inset to Fig. 6(a) of the main text) is not quite as pronounced in the sample discussed here (see inset). At present, the source of these differences has not been revealed. Sample dependences, however, cannot be excluded.

V. HALL MEASUREMENTS

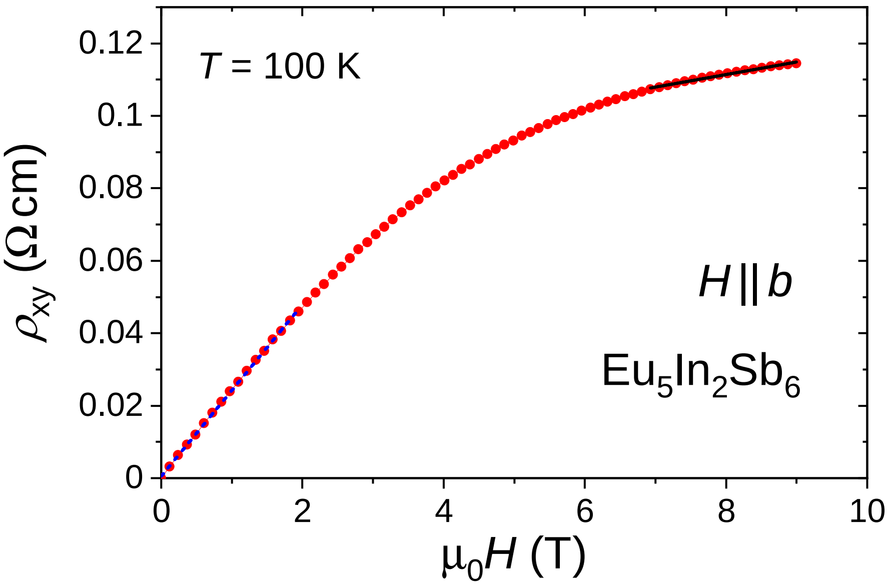

FIG. S6. Hall resistivity ρ_{xy} (red circles) of a single crystal $Eu_5In_2Sb_6$ for $H \parallel b$ and at $T = 100$ K. The original magnetotransport data were measured for positive and negative magnetic fields, the Hall voltage was then obtained as the asymmetric component under field reversal. The black line is a linear fit of the $\rho_{xy}(H)$-data within the range 7 T $\leq \mu_0 H \leq$ 9 T, yielding a slope of about $3.5 \cdot 10^{-5}$ $\Omega\,$m/T. Also included is, for comparison, a fit to the low-field data, $\mu_0 H \leq 2$ T (blue dashed line), with a slope of $2.4 \cdot 10^{-4}$ $\Omega\,$m/T.
